# Cloning and overexpression of a new chitosanase gene from *Penicillium *sp. D-1

**DOI:** 10.1186/2191-0855-2-13

**Published:** 2012-02-16

**Authors:** Xu-Fen Zhu, Hai-Qin Tan, Chu Zhu, Li Liao, Xin-Qi Zhang, Min Wu

**Affiliations:** 1 College of Life Sciences, Zhejiang University, Hangzhou 310058, P. R. China

**Keywords:** Chitosanase, *Penicillium *sp., Gene cloning, Expression

## Abstract

A chitosanase gene, *csn*, was cloned from *Penicillium *sp. D-1 by inverse PCR. The cDNA sequence analysis revealed that *csn *had no intron. The deduced CSN protein consists of 250 amino acids including a 20-amino acid signal peptide, and shared 83.6% identity with the family 75 chitosanase from *Talaromyces stipitatus *(B8M2R4). The mature protein was overexpressed in *Escherichia coli *and purified with the affinity chromatography of Ni^2+^-NTA. The novel recombinant chitosanase showed maximal catalytic activity at pH 4.0 and 48°C. Moreover, the activity of CSN was stable over a broad pH range of 3.0-8.0, and the enzymatic activity was significantly enhanced by Mg^2+ ^and Mn^2+^. The CSN could effectively hydrolyze colloidal chitosan and chitosan, while could not hydrolyze chitin and carboxymethylcellulose (CMC). Due to the particular acidophily, CSN has the potential application in the recycling of chitosan wastes.

The GenBank/EMBL/DDBJ accession numbers for the 18S rRNA gene and chitosanase gene of strain D-1 are JF950269 and JF950270, respectively.

## Introduction

Cellulose, chitin, and chitosan are all composed of β-1,4-linked glucopyranoses, and the difference is in functional groups at the C-2 positions of their constituent sugars, i.e., the hydroxyl, acetamido, and amino groups, respectively. Chitin is a linear homopolymer composed of β-(1,4)-*N*-acetyl-D-glucosamine (GlcNAc), while chitosan is a polycationic carbohydrate consisting of β-(1,4) linked D-glucosamine (GlcN) residues and derived from chitin by partial or complete deacetylation. Chitosan could be found only in fungi cell wall and insect cuticle of limited groups in nature. Chitosan and the products derived from its hydrolysis have attracted much attention because of their interesting biological properties, such as the antibacterial, antifungal and antitumor functions, and thus have been used in agriculture, food and pharmaceutical industries (Somashekar and Joseph 1996; Chiang et al. 2003).

Chitosanase (EC 3.2.1.132) is a member of glycosyl hydrolase families acting on the β-1,4-glycosidic linkage of chitosan to release chito-oligosaccharides. Chitosanase is regarded to be important in carbon and nitrogen recycles that extensively occur in nature, and useful in the preparation of biofunctional chitooligosaccharides. It has been concluded that chitosanases can hydrolyze GlcN-GlcN, GlcN**-**GlcNAc and GlcNAc-GlcN bonds except GlcNAc-GlcNAc bond (Fukamizo et al. 1994; Masson et al. 1994).

Chitosanases have been found from many kinds of organisms, such as bacteria (Somashekar and Joseph 1996; Zhang et al. 2001; Kimoto et al. 2002; Gupta et al. 2010), fungi (Jung et al. 2006; Cheng et al. 2006), viruses (Sun et al. 1999) and plants. Based on amino acid sequence similarity (Henrissat and Davies 1997), chitosanases can be grouped into five glycosyl hydrolase (GH) families, i.e. GH5, GH8, GH46, GH75 and GH80 (http://www.cazy.org/Glycoside-Hydrolases.html). Most bacterial chitosanases belong to family GH46 (Henrissat and Bairoch 1996), while some from fungi belong to family GH75 (Cheng et al. 2006). Chitosanases from family GH5 and GH8 also tend to have β-1, 4-glucanase activity. In contrast, chitosanases of family GH46, GH75, and GH80 are more specific in substrate, which is limited to chitosan (Bueren et al. 2009). Most bacteria can be induced by exogenous chitosan to expression chitosanase and thus play an important role in degradation of this polysaccharide. By now, only a few fungal chitosanases have been researched, which were from a limited fungi strains. Their physiological functions are still unclear, and the gene cloning and expressing are seldom reported. It is of interest to characterize the gene structure of fungal chitosanase in relation to their catalytic function and compare this relationship between fungal and bacterial chitosanases.

A chitosanase-producing fungus, *Penicillium *sp. D-1, was isolated. Here, we describe the cloning of a new chitosanase gene, its overexpression in inclusion bodies, and protein refolding. The novel recombinant chitosanase exhibited chitosan-hydrolyzing activity.

## Materials and Methods

### The chitosanolytic strain and identification

The fungal strain D-1 was isolated using modified Czapek-Dox medium, containing 0.2% NaNO_3_, 0.1% K_2_HPO_4_, 0.05% MgSO_4_, 0.05% KCl, 0.001% FeSO_4 _and 0.5% colloidal chitosan, pH 5.0, in which chitosan was the sole carbon source. Culture cultivating for 5 days was used to prepare chromosomal DNA. The total RNA extraction was performed using cells growing in chitosan medium for 4 days at 30°C. PCR amplification of 18S rRNA gene was carried out using the primers FP4 and RP1438, which correspond to the position 4-18 and 1420-1438 of that of *Saccharomyces cerevisiae*, respectively (Table 1). Sequencing was performed at Genscript Co., Ltd (Nanjing, China).

**Table 1:** Primer pairs of synthetic oligonucleotide designed

Primer	Oligonucleotide sequence
FP4	5'-CTGGTTGATYCTGCCAGT-3'
RP1438	5'-GGGCATCACAGACCTGTTAT-3'
DFP	5'-AAYATGGAYATHGAYTGYGA-3'
DRP	5'-RTCDCCCCADATNCCRTA-3'
FP1	5-CCAGAGCACGTTGGCATCAA-3
RP1	5-ACCATAGTCGGACTTGACCT-3
FP2	5'-GGTCTGCAACAACAAGCTCATC-3'
RP2	5'-GAGTCGATGCCGTCTTGATC-3'
csn -f	5'-CGCCATATGAAAACAGCTGCCATT-3
csn -F	5'- CGCCATATGTACGATATCCCTGACAAC-3'
csn -r	5'-ATTCTCGAGTTACAGAGACGCAACAAG

The primers, csn-f and csn-F were designed with a *Nde*I site, while csn-r was with *Xho*I site, which are underlined are underlined, respectively.

### Extraction of chromosomal DNA and gene cloning of chitosanase by I-PCR

The chromosomal DNA of strain D-1 was extracted by the phenol-chloroform method (Chomczynski and Sacchi 1987). Based on the highly conserved regions of fungal chitosanase, two degenerated primers (Table 1 DFP and DRP) were designed to amplify the partial chitosanase (*csn*) gene. Then, the 5' and 3' flanking regions of partial *csn *gene were cloned by inverse PCR according to Ochman *et al*. (1988). The chromosomal DNA (5 μg) was completely digested with *Pst*I, *Bam*HI, *Eco*RI and *Hin*dIII, respectively. The digested fragments were self-ligated to form circular DNAs with T4 DNA ligase (Takara Bio) at 16°C overnight. The ligated DNAs were purified and subjected to serve as a template for inverse PCR. The first PCR was performed using primers FP1 and RP1 (Table 1), which was designed according to the sequence of the partial *csn *gene by Primer Premier 5. A nested PCR was performed further using primers FP2 and RP2 (Table 1) with the first PCR product (diluted 100-1000 times) as the template. The secondary PCR product was purified and inserted into pMD19-T vector (TaKaRa Cloning kit) for sequence analysis.

### Extraction of RNA and RT-PCR

Total RNA was extracted from the mycelia of strain D-1 using TRIZOL Reagent (Invitrogen). Full-length chitosanase cDNA was obtained from total RNA by RT-PCR using a reverse transcription kit (TaKaRa). PCR amplification was performed using primers csn-f and csn-r (Table 1). The PCR product was sequenced after TA clone as described above.

### Sequence analysis and phylogenetic tree

Obtained *csn *sequence was analyzed for the identity and similarity with other related sequences by BLAST on-line. The signal peptide was deduced by SignalP 3.0 Server (http://www.cbs.dtu.dk/services/SignalP/). The phylogenetic analysis was performed among amino acid sequences of known chitosanase using Clustal X program (Jeanmougin *et al*. 1998) and MEGA 4 program package (Tamura *et al*., 2007).

### Expression and purification of recombinant chitosanase

To characterize CSN chitosanase, primers csn-F and csn-r (Table 1) were used to amplify the mature chitosanase gene, which was cloned into the vector pET28a. The resulting vector, pET28a-*csn*, was transformed into *E. coli *BL21 (DE3) and grown in Luria-Bertani medium (containing 20 μg/mL kanamycin) at 37°C. After an OD_600 _of 0.6-0.8 was reached, it was induced by 0.1 mM of isopropyl-D-thiogalactopyranoside (IPTG) at 30°C for 5 h. The host cells of 1.5 ml culture were collected, and then were sonicated with PBS buffer added. After centrifugation, the protein of the collected cells and the supernatant were analyzed by SDS-PAGE. The rest of host cells were collected, and resuspended in cold STE buffer (50 mM Tris, pH 8.0, 1 mM EDTA, 150 mM NaCl, 5% glycerol, v/v) and treated with 0.1 mg/ml lysozyme for 40 min on ice. β-Mercaptoethanol was then added up to the final concentration of 0.01%, followed by the addition of sarkosyl (Sangon, China) at a concentration of 0.3% (v/v). After the pretreatment described above, the cell suspension was sonicated at a low power (20 W; 1 s sonicating vs. 2 s pause) for 30 min. After centrifugation (18,000 g, 20 min, 4°C), the supernatant was amended with Triton X-100 at a concentration of 0.5% (v/v) and mixed gently for 30 min at room temperature (Cao *et al*. 2008), and then loaded onto an affinity chromatography column filled with Ni^2+^-NTA (Qiagen). Before the His-CSN fusion protein was eluted by elution buffer (100 mM imidazole, 0.5 M NaCl, 50 mM PBS, pH 8.0), the column was washed extensively with wash buffer (20 mM imidazole, 0.5 M NaCl, 50 mM PBS, pH 8.0) to remove unspecifically bound proteins. The purified protein was stored at -80°C until used.

### Electrophoresis and chitosanase activity assay

The purified CSN enzyme was analyzed by SDS-PAGE, and protein concentration was determined by Bradford method. The chitosanase activity was assayed as described previously by measuring the amount of reducing sugar product with colloidal chitosan as the substrate (Zhu et al. 2007). The reaction mixture were consist of 0.5 ml enzyme solution and 0.5 ml of 1% colloidal chitosan in 1 ml McIlvaine buffer at the indicated pH. The mixture was incubated for 30 min at 48°C, and the reaction was stopped in boiling water for 10 min. The amount of reducing sugars released in the supernatant was measured by a method that uses dinitrosalicylic (DNS) acid reagent, and the absorbance was measured at 540 nm. 1 unit (U) of chitosanase activity was defined as the amount of enzyme that liberates 1 μmol of detectable reducing sugar at 48°C from the substrate per min with GlcN as the standard.

### Effect of pH and temperature on activity and stability of chitosanases

Chitosanase activity was measured at the pH range of 2.5 to 8.0 for 30 min before assayed by standard assay method. The residual chitosanase activity was also measured after the enzyme was incubated at certain pH at 37°C for 60 min.

The effect of temperature on enzyme activity was determined by incubating the reaction mixture at different temperatures for 30 min before assayed by the standard method. The thermostability of the protein was examined by measuring remaining activity after the enzyme was incubated at certain temperature for 60 min.

### Effect of metal ions and substrate specificity

Various metal ions in the form of chloride salt, such as Na^+^, K^+^, Mg^2+^, Ca^2+^, Co^2+^, Mn^2+^, Fe^3+^, Cu^2+^or Ag^+ ^(AgNO_3_), were added into reaction mixture at the final concentration of 10 mM. The corresponding activities were assayed by the standard method.

Chitosanase activity was measured by the standard method with different substrates (at 1% concentration), such as chitosan, colloidal chitosan, colloidal chitin and carboxymethylcellulose (CMC).

## Results

### *Penicillium *sp. D-1 identification

A fungal strain D-1 which formed clear hydrolysis zone on Czapek-Dox plate was isolated from soil. According to the analysis of its partial 18S rRNA gene sequence (accession number JF950269), strain D-1 was most closely related to the species of *Penicillium *with the similarity over 99%, and strain D-1 showed characteristic brush hypha of *Penicillium*. Thus, the isolated strain D-1 was identified as *Penicillium *strain D-1 and deposited to China General Microbiological Culture Collection Center (CGMCC), and the strain number: CGMCC 3.15301.

### Chitosanase gene cloning

A single fragment of 270 bp was amplified using degenerate primers DFP and DRP from the genomic DNA of *Penicillium *sp. D-1. The deduced amino acid sequence of this fragment showed a significant similarity (50%-60%) to the fungal chitosanases from *Fusariym solani *f. sp. *phaseoli*, *Beauveria bassiana*, *Metarhizium anisopliae *var. *acridum *and *Aspergillus oryzae *strain IAM 2660.

A fragment of 2430 bp, including a chitosanase gene and its flanking regions, was finally obtained by inverse PCR. The G+C content of the open reading frame is 55.8 mol%. The cDNA corresponding to the whole ORF was further amplified by RT-PCR. The *csn *gene contained 753 bp, encoding 250 amino acids with a putative signal peptide of 20 amino acid residues (accession number JF950270). The mature CSN has a predicated molecular mass of 24.6 kDa and a deduced pI value of 4.18.

**Figure 1:**
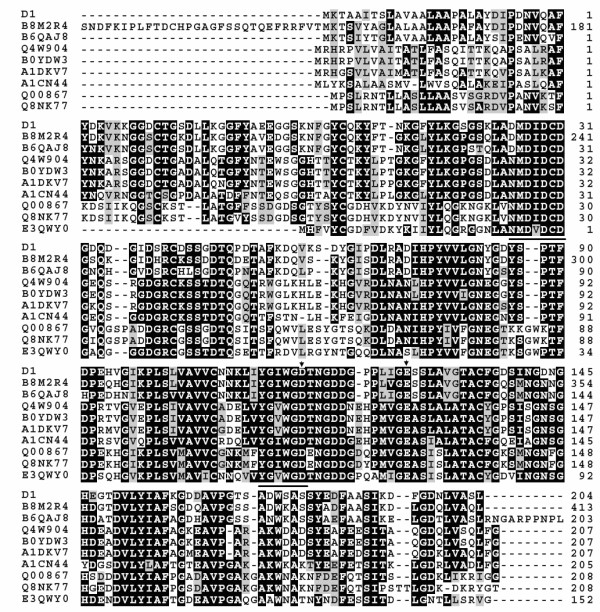
**Multi-alignment of fungal chitosanases**. The deduced amino acid sequences from 9 genes sequences were aligned by Mega 4. Amino acids that are identical between CSN and other sequence are shaded in black and the two conserve regions are underlined. The residue that was not identical but at least similar to the column-consensus be printed in another user-defined rendition usually on gray background. Vertical arrows indicate essential amino acid residues for catalytic activity of fungal chitosanases. GenBank accession details are for *Penicillium *sp. D-1 (this study), *Talaromyces stipitatus *(B8M2R4), *Penicillium marneffei *(B6QAJ8), *Aspergillus clavatus *(A1CN44), *Neosartorya fischeri *(A1DKV7), *N. fumigata *(Q4W904), *A. fumigatus *(B0YDW3), *Fusarium solani *subsp. *Phaseoli *(Q00867), *F. solani *f. *robiniae *(Q8NK77) and *Colletotrichum graminicola *(E3QWY0).

Homology analysis of amino acid sequence showed the high similarity between CSN and those fungal chitosanases (Figure 1) from *Talaromyces stipitatus *ATCC 10500 (B8M2R4, 83.6%), *Penicillium marneffei *ATCC 18224 (B6QAJ8, 80.8%), *Aspergillus clavatus *(A1CN44, 54.6%), *Neosartorya fischeri *NRRL 181 (A1DKV7, 54.2%), *N*. *fumigata *Af293 (Q4W904, 53.4%), *A. fumigatus *A1163 (B0YDW3, 53.0%), *F. solani *subsp. *Phaseoli *(Q00867, 49.0%), *F. solani *f. *robiniae *(Q8NK77, 49.6%) and *Colletotrichum graminicola *(E3QWY0, 54.6%).

### Purification of CSN

**Figure 2:**
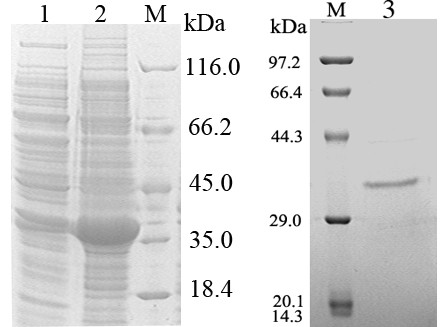
**SDS-PAGE analysis of the recombinant CSN overexpressed in *E. coli *BL21 (DE3)**. Lane M, protein size markers. The sizes of protein markers are indicated. Lane 1, the supernatant of sonicated cell suspension. Lane2, the total cell lysate. Lane 3, purified CSN by affinity chromatography of Ni^2+^-NTA.

The cloned *csn *gene was overexpressed in *E. coli*, and the recombinant CSN was in the sediment of sonicated cell suspension (Figure 2, Left). The recombinant CSN was purified using Ni^2+^-NTA column after the denatured condition and then refolded. Its apparent molecular mass determined by SDS-PAGE was about 36 kDa (Figure 2, Right). The purified protein showed chitosan-hydrolyzing activity (26.4 U/mg).

### Effect of pH and temperature on chitosanase activity

**Figure 3:**
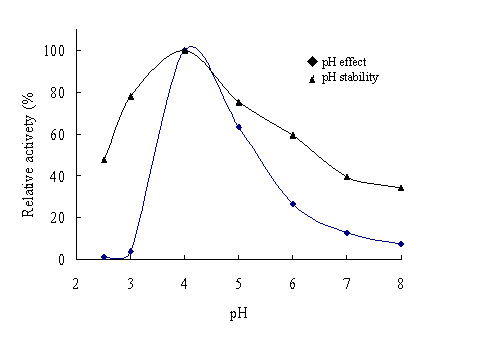
**Effects of pH on the activity (◆)**. The enzyme activities were measured under standard assay conditions with various buffers at pH 2.5 to 7.0. Stability of the chitosanases (▲). The enzymes were incubated for 60 min at 37°C in various buffers at pH 2.5 to 8.0 and the residual activities were assayed under standard conditions. The buffers used were McIlvains buffer. The highest residual chitosanase activity, which was dealt with after 1 h at 37 degC at pH4, was setted 100%.

**Figure 4:**
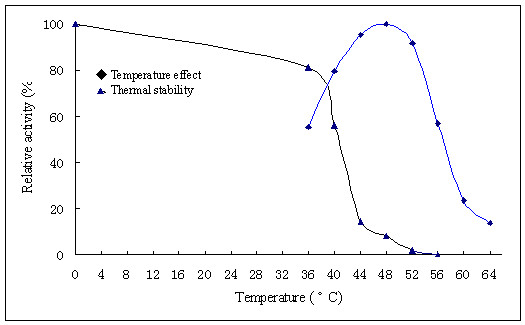
**Effects of temperature on the activity (◆)**. The enzyme activities were assayed under standard conditions at various temperatures from 36 to 64°C. Stability of the chitosanases (▲). The enzymes were incubated for 60 min at various temperatures and the residual activities were assayed under standard conditions. The highest residual chitosanase activity, which was dealt with after 1 h at 0 degC at pH4, was setted 100%.

CSN optimally hydrolyzed chitosan at pH 4.0. When CSN was kept in McIlvains buffer in the pH range of 2.5 to 8.0 at 37°C for 1 h, the CSN activity was relatively stable at pH 3.0-5.0 (Figure 3). CSN had an optimal temperature for catalyzing of 48°C. However, enzyme is unstable at 48°C by losing 92% activity after incubated for 1 hour (Figure 4).

### Metal ions on chitosanase activity

Mn^2+ ^and Mg^2+ ^stimulated the enzyme activity significantly. With colloidal chitosan as substrate, the activity of CSN was increased about 1.9 and 1.4 fold when Mn^2+ ^and Mg^2+ ^existed, respectively. However, the enzyme activity was strongly inhibited by Fe^3+^, Ag^+ ^and Cu^2+ ^(Data not shown).

### Substrate specificity of chitosanase

The specificity of the purified enzyme on various substrates was tested. CSN hydrolyzed colloidal chitosan and chitosan effectively. However, no obvious activity was detected when colloidal chitin and carboxymethylcellulose was used as substrates.

## Discussion

Based on the phenotypic characteristics and 18S rRNA gene sequence analysis, the fungus was identified as a member of the genus *Penicillium*. Up to now, other reported *Penicillium *strains which could produce chitosanase were *P. islandicum*, *P. spinulosum *and *P. chrysogenum *(Fenton and Eveleigh, 1981; Ak *et al*., 1998; Rodríguez-Martín et al, 2010).

Two highly conserved regions, NMDIDCD and YGIWGD were found from GH75 family chitosanases according to the alignment of the deduced amino acid sequences of some fungal chitosanases. Consequently, a pair of degenerate primers, DFP and DRP were designed to amplify a single fragment of 270 bp, and then the fully *csn *gene fragment was further cloned by I-PCR. The deduced amino acid sequences DIDCD of CSN were 100% similar to the corresponding conserved region of fungal chitosanases which have been classified into family GH75. It should be noted that CSN showed no similarity to bacterial chitosanases, suggesting a different evolutionary origin between fungal chitosanase and bacterial counterpart.

**Figure 5:**
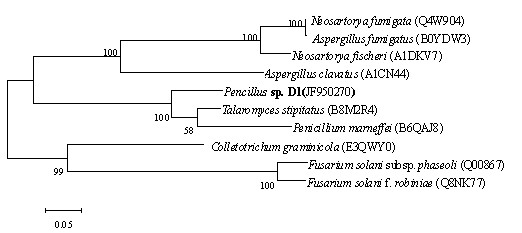
**Phylogenetic sequences of GH75 chitosanases from 10 fungal proteins**. The Phylogenetic tree was constructed with Mega 4. Neighbor-joining was used with 1000 random bootstrap replication. Alignment of the GH75 domain amino acid sequences used Clustal X (1.83). A bar represents the evolutionary distance of 0.05. The DDBJ/EMBL/GenBank accession numbers are shown in parentheses. The multiple sequence alignment was performed using Clustal X program (Jeanmougin *et al*. 1998) and visually examined with BoxShade Server program (http://www.ch.embnet.org/software/BOX_form.html).

Although CSN exhibited high homology with the chitosanases of *T. stipitatus *ATCC 10500 and *P. marneffei*, they were different in molecular weight. CSN of *Penicillium *sp. D-1 consists of 250 amino acids, while the chitosanases of *T. stipitatus *ATCC 10500 and *P. marneffei *ATCC 18224 were composed of 459 amino acids and 1070 amino acids, respectively. As shown in the phylogenetic tree of the GH75 chitosanases (Figure 5), CSN of *Penicillium *sp. D-1 was different from the chitosanases of *T*. *stipitatus *and *P. marneffei *by clustering into the outlying clade of orthologous chitosanases.

To ensure that the cloned *csn *gene could encode a functional chitosanase, the *csn *gene encoding the mature protein without the signal sequence was heterologously overexpressed in *E. coli*. As shown in Figure 2, expression of the CSN chitosanase resulted in large amounts of insoluble recombinant protein, and Sarkosyl and Triton-100 were used to solubilize the inclusion body. The Sarkosyl is thought to help solubilize the protein by partially denaturing it, and the subsequent addition of Triton-100 allows the renaturation of the protein. The active CSN was purified using Ni^2+^-NTA column after the denatured condition and then refolded. This is a simple and efficiently purification methods for active protein from inclusion body.

Although the calculated molecular mass of the mature protein was 24.6 kDa, the approximate size of the His-tagged protein was 36 kDa as showed by the SDS-PAGE electrophoresis. This discrepancy is apparently related to the acidic nature of the protein (16.9% glutamate and aspartate compared to 8.7% lysine and arginine and histidine). The predominance of acidic residues in most proteins results in their abnormal behavior during SDS-PAGE (Izotova et al., 1983).

As previously reported, chitosanase from *P. islandicum *was a moderately acidiphilic enzyme, with the pH optimum at 4.5 to 6.0 and optimum temperature at 45°C. The optimum catalyzing condition for chitosanase from *P. spinulosum *is pH 5.0 and 55°C. Correspondingly, *Penicillium *sp. D-1 produced an acidiphilic chitosanase, showing maximum activity at pH 4.0 and 48°C. Thus, it is concluded that the chitosanase CSN of strain D-1 prefers lower pH and higher temperature to optimally hydrolyze substrate.

In conclusion, a novel gene belonging to GH75 chitosanase was cloned from a newly isolated *Penicillium *sp. D-1, and was successfully expressed in *E. coli *BL21 (DE3). To our knowledge, this is the first report on expression and characterization of *Penicillium *chitosanase. Biochemical and molecular characteristics showed that CSN should be a novel chitosanase which could be considered as a potential candidate for producing chitooligosaccharides from chitosan.

## Abbreviations

CMC: carboxymethylcellulose; GH: glycosyl hydrolase; *csn*: chitosanase; GlcNAc: *N*-acetyl-D-glucosamine; GlcN: D-glucosamine; IPTG: isopropyl-D-thiogalactopyranoside

## Competing interests

The authors declare that they have no competing interests.
